# Constructing a database for the relations between CNV and human genetic diseases via systematic text mining

**DOI:** 10.1186/s12859-018-2526-2

**Published:** 2018-12-31

**Authors:** Xi Yang, Zhuo Song, Chengkun Wu, Wei Wang, Gen Li, Wei Zhang, Lingqian Wu, Kai Lu

**Affiliations:** 1 School of Computer Science, National University of Defense Technology, Changsha, 410073 China; 2 Genetalks Biotech Inc., Beijing, 100176 China; 3 Institute for Quantum Information & State Key Laboratory of High Performance Computing, College of Computer, National University of Defense Technology, Changsha, 410073 China; 4 Center for Medical Genetics, Central South University, 110 Xiangya Road, Changsha, 410078 Hunan China

**Keywords:** Copy number variant (CNV), Disease, Named entities recognition, Relation extraction, Parallel computing

## Abstract

**Background:**

The detection and interpretation of CNVs are of clinical importance in genetic testing. Several databases and web services are already being used by clinical geneticists to interpret the medical relevance of identified CNVs in patients. However, geneticists or physicians would like to obtain the original literature context for more detailed information, especially for rare CNVs that were not included in databases.

**Results:**

The resulting CNVdigest database includes 440,485 sentences for CNV-disease relationship. A total number of 1582 CNVs and 2425 diseases are involved. Sentences describing CNV-disease correlations are indexed in CNVdigest, with CNV mentions and disease mentions annotated.

**Conclusions:**

In this paper, we use a systematic text mining method to construct a database for the relationship between CNVs and diseases. Based on that, we also developed a concise front-end to facilitate the analysis of CNV/disease association, providing a user-friendly web interface for convenient queries. The resulting system is publically available at http://cnv.gtxlab.com/.

## Background

A human copy number variant (CNV) is a DNA segment greater than one kilo base (kb) differing from two copies. CNVs could cause diseases by gene dosage, gene disruption, gene fusion, or position effects. The detection of CNVs and interpretation of their medical significance are a routine in several genetic tests. [1, 2]. Many online databases and web search services for CNVs in normal and/or disease populations have been developed to facilitate the interpretation of CNVs in clinical settings, such as Database of Genomic Variants (DGV), The International Standards for Cytogenomic Arrays (ISCA) Consortium, DECIPHER and ClinGen. Despite cytogeneticists or physicians could use these databases to interpret the clinical test results, they still need to always search and read the original literature for rare CNVs from PubMed. Empowered by text mining techniques, CNV-GT provides a convenient interface for fast and automated queries about reported CNVs in PubMed articles, as well as diseases contexts and important literature information. The original context which states the CNV/disease relationship is displayed and highlighted.

### Related work

In recent years, biomedical knowledge has been growing rapidly, most of which are presented in the form of curated databases and scientific literature.

Curated databases are one type of tools for researchers or health professionals to obtain needed knowledge. For example, ClinGen (https://www.clinicalgenome.org/), Database of Genomic Variants (DGV: http://dgv.tcag.ca/dgv/app/home), and DECIPHER (https://decipher.sanger.ac.uk/) are the most comprehensive databases to aggregate information about the relationship between genomic variations and human health conditions. Based on the earlier International Standards for Cytogenomic Arrays Consortium (ISCA), ClinGen [3] was initiated as a National Institute of Health funded program. Now it includes CNV data from microarray testing and data on sequence variants from clinical molecular testing. In contrast to ClinGen, DGV only collects structural variations identified in healthy control samples [4]. Different from ClinGen and DGV, the DECIPHER database collects data from patients (> 20,000) who have given consent for broad data-sharing and shares these data with the clinical community to interpret the relationship between genomic variations and health conditions [5]. In practice, the coverage of curated databases is limited, as curation is a laborious and time-consuming process. Therefore, we often need to resort to literature to search for supporting evidences.

Scientific literature, one of the most important sources of biomedical knowledge, is accumulating explosively. For instance, there are over 28 million MEDLINE abstracts and 4.9 million PMC full-texts available. Such a massive amount of information is mostly presented as unstructured texts, which makes it difficult for any expert to digest that huge amount of knowledge within a reasonable period of time. Automated/semi-automated tools are essential for enabling efficient accesses to structured and searchable biomedical knowledge.

Text mining methods enable automated and systematic analyses of literature [6, 7]. Many techniques were developed to assist information retrieval, information extraction, database development and hypothesis generation [8]. The major aim of text mining is to identify potentially useful information in the literature and present it in a structured way. Text mining has demonstrated its potential in boosting biocuration and biomedical knowledge collection [9–12]. We have seen successful applications on named entity recognition (NER) for genes/proteins [13, 14], diseases [15], species [16], mutations [17], chemicals [18], etc. In this paper, two types of concepts needing NER and normalization are diseases and CNVs. For diseases, advanced tools like DNorm [15] and TaggerOne [19] are available for NER and normalization. Particularly, DNorm is a state-of-the-art general purpose toolkit for NER and normalization, with a NER f-score of 0.782 and a normalization f-score of 0.809. For CNVs, however, no dedicated NER tool exists to date.

Currently, the methods for extracting relationships can fall into several categories: co-occurrence [20] methods, pattern or rule based methods, and machine learning methods, or hybrid methods are also available.

The co-occurrence method [21] means that if two entities appear in the same paragraph of text, the two entities are considered related. This method is fast and simple, but achieves a high recall rate at the expense of accuracy and does not provide detailed relationship attributes between entities.

The rule-based method [22–24] is mainly to use the information contained in the lexical and grammatical phenomena to predict the relationship. Typical characteristic information includes verbs, nouns, prepositions, and the like. Rule-based method accuracy is generally high, and it is often possible to obtain detailed relationship attributes between entities. However, the formulation and generation of rules often rely on a large number of annotation collections and rich experience of human experts.

The method based on machine learning [25–27] mainly uses a certain number of labeled documents as training data sets to extract features of words or sentences, classifies them by machine learning models, and determines the relationship categories between two target entities (or No relationship). However, the acquisition of marker data for establishing ML models requires many human experts to participate in labeling, which consumes labor and financial resources.

For CNVs and diseases relationship, Qiu et al. published the Copy Number Variation in Disease (CNVD) database [28]. Although the paper has “text mining-based” in its title, CNVD were actually built by manually extracting information from 6301 published papers. CNVD includes associated diseases, genes, chromosome segments, and the descriptions of CNVs by linking information from the NCBI Gene and Gene Ontology databases. To date, no automated text mining tool was published on revealing the CNV-disease relationship.

It is time consuming to process a large number of articles with one single machine or a small cluster of servers. Therefore, it is necessary to harness the power of high performance computing. For instance, in BioContext [29], Gerner et al. employed 100 concurrent processes and managed to finish the processing of the full MEDLINE and PMC Open-Access dataset within 3 months. Wu et al. carried out parallel text mining using Tianhe-2 supercomputer with a scalable pipeline [30]. to a higher F-score.

## Methods

### Data sources

The webserver backend is populated with data from multiple sources. Standard disease names and the corresponding MeSH IDs or OMIM IDs are obtained from the CTD database [31]. CNV related articles are retrieved from a local copy of the NCBI MEDLINE 2018 baseline based on a list of PubMed IDs, which are obtained by posting a carefully composed query of CNV to PubMed online. The local literature database is constructed by parsing the MEDLINE 2018 baseline XML files.

### Text mining procedure

**Fig. 1:**
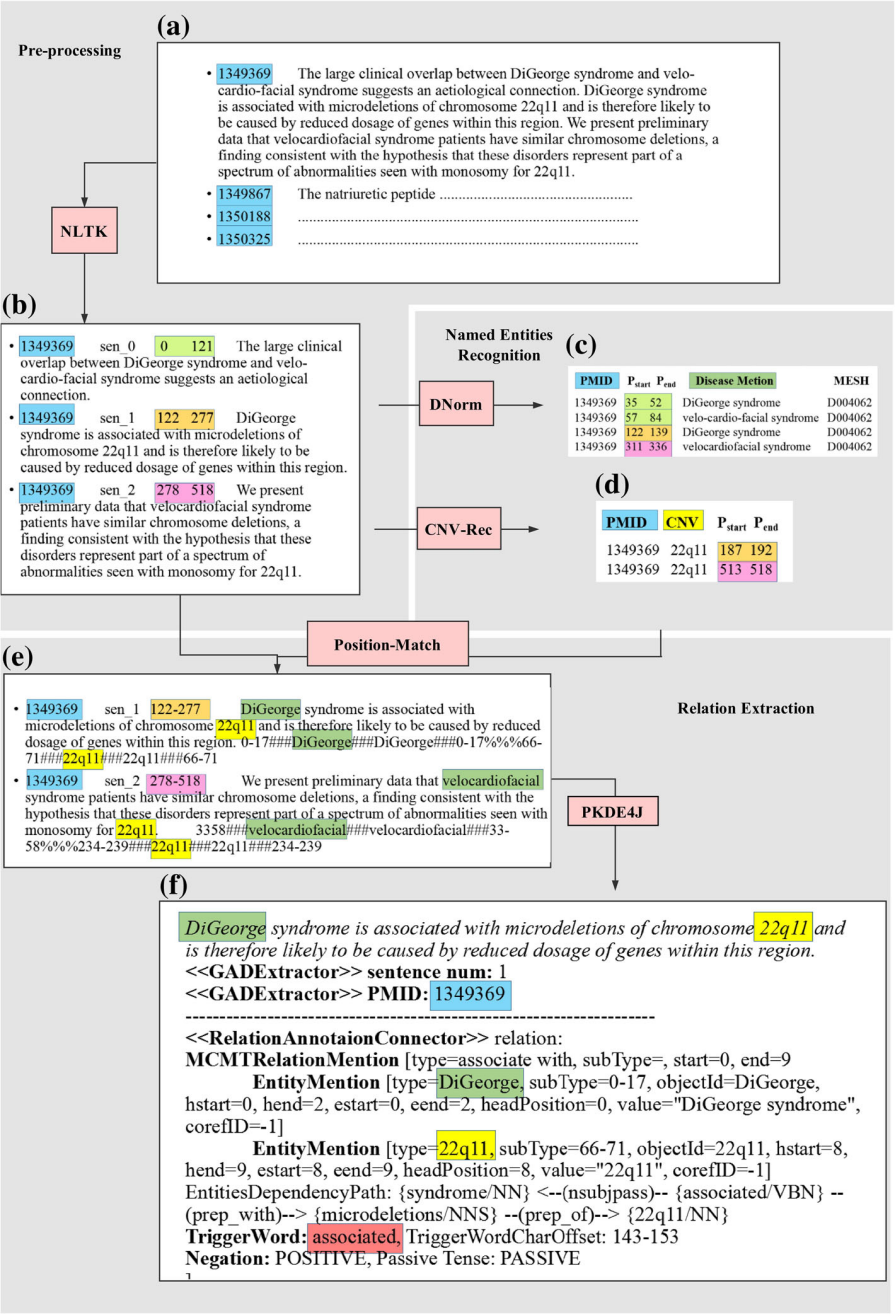
The workflow of text mining. **a** Initial abstracts file (**b**) The result of using NLTK to split abstracts into clauses (**c**) The result of using DNorm to recognize disease entities. **d** The result of using CNV-Rec to recognize CNV entities. **e** match the location of CNV and disease in sentences. **f** The result of using PKDE4J to extract the relation between CNV- disease. *NLTK:Natural language toolkit, a set of Natural Language Processing tools based on python. It can be used for text categorization, symbolization, root extraction, labeling, parsing, semantic reasoning, or packaging into an industrial-grade natural language processing library. *Dnorm:a toolkit of disease name normalization with pairwise learning to rank. *PKDE4J: a toolkit of relation extraction with rules. *CNV-Rec: a regular expressions-based method of CNV recognitionEmbedding feature layer

Three text mining steps are carried out on each article within the corpus, including pre-processing, named entities recognition (NER), and relation extraction (RE). Figure 1 shows the whole workflow of text mining process. Details of each step are explained as follows:

#### Pre-processing

The relationship between CNVs and diseases within one single sentence are considered in our work. Therefore, in the pre-processing stage, unstructured texts are firstly split into separated sentences via NLTK. Then part-of-speech tagging is performed on each sentence, followed by syntactical parsing to produce a grammatical representation of each sentence [7]. An example of sentence splitting is shown in the Fig. 1b.

#### Named entities recognition (NER)

##### Recognizing **disease** mentions in titles and abstracts

Disease mentions are located and normalized by a state-of-the-art tool for disease name extracting, the DNorm system [15], which is implemented using machine learning approach. As shown in Fig. 1c, normalized disease mentions are annotated with MeSH IDs and OMIM IDs (the dictionary is obtained from MEDIC). Based on pairwise learning to rank, DNorm creates a mathematically principled framework for learning similarities between disease mentions and concept names. If a word group matches, it is mapped to the appropriate MEDIC concept names.

##### Recognizing **CNV** mentions in titles and abstracts

The CNVs consist of autosomal mutations and sex chromosome variations. There are two types of autosomal variants: (1) deletion or amplification of the long arm or broken arm region of chromosomes 1 to 22, such as 22q11.2. (2) multiples of chromosome 1 to 22, such as trisomy 21. The major variation in sex chromosome variation is the double of the X chromosome. For example, XXY and XXX. As there are some specific rules to the CNV names, we design several patterns to capture them by using regular expressions. Similarly, the polarity descriptions (duplication/deletion) can also be detected by regular expressions. The specific designed patterns are as follow:

(([1–9]\d?|[xyXY])[pqPQ] [1–9]\d?([\-\~]?[pqPQ]? [1–9]\d?){0,}(\. [1–9]\d{0,1})*)

([tT]risomy\s?([1–9, 0–9]?|x)*)’, r’\s[xX][xX][xX]\s.

r’\s[xX][xX][yY]\s.

The example of CNV recognition is shown in (Fig. 1d).

#### Relation extraction (RE)

After named entities recognition, an operation of position comparing between sentences and entities is performed to generate the instances that consists of two candidate entities within one single sentence, as shown in Fig. 1e.

For each instance, we use a highly flexible and extensible framework, named PKDE4J [22], to identify the relation between the targeted CNV and disease. Based on the dependency parsing for the sentence, the PKDE4J defines a set rules about collocations, logical semantic relationships and dependency path to extract the trigger word. For instance, as shown in Fig. 1f, the example of relation extraction result shows that the trigger word between “DiGeorge” and “22q11” in the sentence is “associated”. Thus, the variation of 22q11 is predicted to be a cause of DiGeorge syndrome.

### Parallel processing

**Fig. 2:**
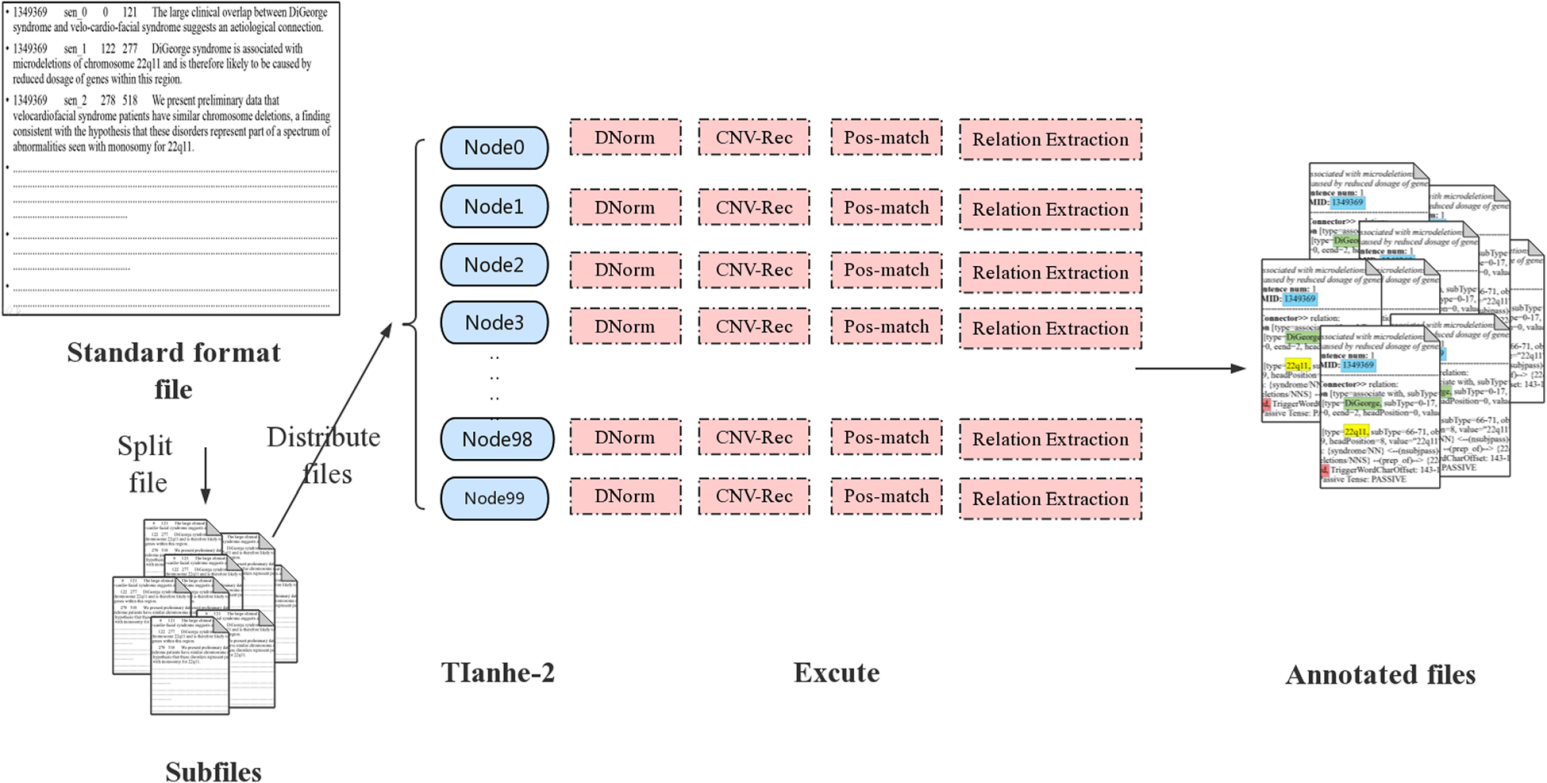
Implementation of parallel processing

Text mining is a computational intensive task. Over 440,000 abstracts are included in our system. To perform text mining efficiently on such a large corpus, we employed parallel processing on the Tianhe supercomputer [30] in a similar way as it was done in Short-Board load balancing algorithm [31]. The basic idea is to split the input into small subsets and process each subset in parallel. A total number of 100 compute nodes were launched, with articles being distributed to each node in a load-balanced manner, that is, the work load of each compute node should be approximately the same. The work load for each node is estimated by the summed length of all allocated articles. There is only one exclusive process on each node as the Dnorm system requires over 40GB per process, while the main memory size of a node is 64GB. It took approximately half an hour to complete the whole processing procedure. More nodes can be introduced to make the processing time even shorter. The parallel text mining framework implemented in this work will be used for future large-scale literature mining for disease-oriented studies. Figure 2 describe the details about the Implementation of parallel processing.

To demonstrate how parallel processing on the Tianhe-2 supercomputer can boost the efficiency of text mining, we evaluated the processing time of 100 files each containing 50 sentences in the process of Relation Extraction. The processing procedure took 4750.4 s using one single node, while on the contrary, it only took 49.74 s by using 100 nodes in concurrent. Therefore, on this example data set, the speed-up is 95.5x by using 100 nodes (almost linear). The whole CNV literature set could take over three days to finish the processing via a rough estimation. This time can be reduced to be less than one hour if we employ enough nodes on Tianhe-2.

Processing the entire CNV literature collection, withing 100 nodes, completed in 3458 s.

### Post-processing (data cleaning and statistics)

To get maximal statistical power, all cohort data are desired, as duplicate values and incorrect values degrade association studies.

We de-duplicate our data after each step of the process to reduce repetitive operations and prevent statistical errors.

The problem of databases containing incorrect values is common in biomedical text mining. This issue arises from various reasons; In named entities recognition, it may be the polysemy. For example, Plasma can represent the fluid composition of blood, and can also represent a key technology in KDE4. In relation extraction, the complex and diverse of semantic structure and the lacking of professional background knowledge databases cause we cannot find the trigger word to classify the relation between CNV and disease. Therefore,we delete the wrong named entities recognition and change a part of wrong relation extraction results manually. Simultaneously, we provide a feedback mechanism. After a user visits, he (or she) can give feedback on the wrong results and we will make timely changes.

Finally, we statistical results. By searching for diseases, count the number of sentences searched and see the number of each type CNVs mentioned in each sentence. According to the order of occurrence of CNVs, the twenty CNVs that are most relevant to the disease are recommended. This operation is also applied to CNVs to obtain the most relevant list of CNV diseases.

### Web implementation

A web front end is implemented in AJAX for dynamic data loading and Canvas for animations. A back-end server is implemented in the Django framework to provide RESTful APIs, which provides data access and operations to the front end.

**Fig. 3:**
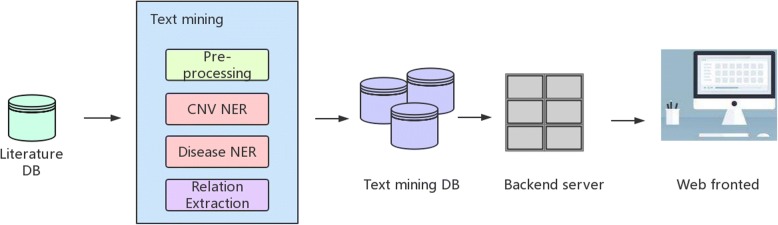
Architecture of CNVdigest

The whole system is implemented in an architectural pattern commonly used for developing user interfaces that called Model–view–controller (MVC). The web front end employs AJAX for dynamic data loading and Canvas for animations. It is written in CoffeeScript/Less. The architecture of the webserver utilizes the Python-based Django framework to provide RESTful APIs, which provides data access and operations to the front-end AJAX code. Databases are implemented in MySQL. Back-end deployment uses Docker + Cirus + gunicorn to ensure the stability and maintainability of our service. The system architecture is illustrated in Fig. 3.

The whole system is based on the text mining of a selected literature subset, with several NLP components involved. Results from text mining are injected into a database for efficient queries, handled by a backend server, which also accepts user inputs received at the web frontend.

### Queries and results presentation

**Fig. 4:**
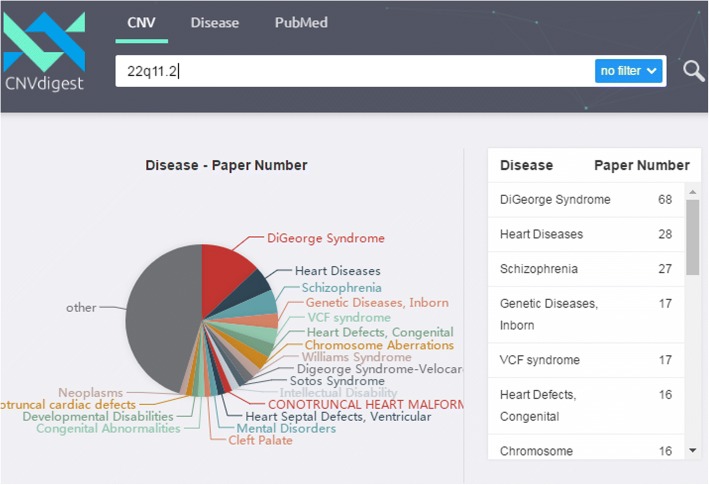
Example searching result of cytoband “22q11.2” in CNVdigest. The disease and numbers of paper found in NCBI were displayed with a pie-chart (left) and a list (right)

**Fig. 5:**
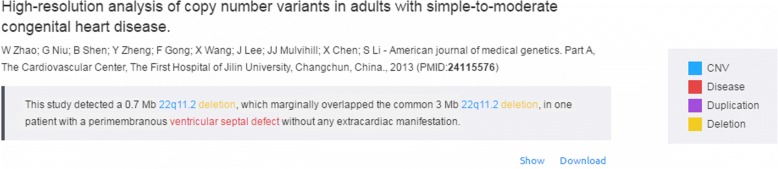
Example evidence sentences of one paper listed below the summary. Multiple evidence sentences within the same article are grouped and the key concepts (CNVs, duplication/deletion, disease names) are highlighted by different colors

The web server provides three different query perspectives to users: 1) input a CNV to find the most relevant diseases as described in literature; 2) input a disease name or select one disease from a given list (indexed by disease MeSH terms) to find related CNVs; 3) input a PubMed article ID (PMID) or a list of PMIDs to find CNV-diseases correlation in those articles. The results include a statistical summary and details of evidences from literature. The summary consists of a pie-chart and a ranking list. For a given CNV, for instance 22q11.2, the top 20 diseases are presented in the pie chart, and a ranking list is also displayed, ordered by the number of PubMed articles with descriptions of diseases correlated to 22q11.2 (Fig. 4). Evidence sentences are listed below the summary. Multiple evidence sentences within the same article are grouped and the key concepts (CNVs, duplication/deletion, disease names) are highlighted with different colors (Fig. 5). The whole annotated abstract for a PubMed article can be viewed via the ‘Show’ link or can be downloaded via the ‘Download’ link.

## Results

The CNVdigest database includes 440,485 sentences for CNV-disease relationship. A total number of 1582 CNVs and 2425 diseases are involved. We tested the utility of CNVdigest by posting CNVs and diseases queries to our system. For instance, a CNV query “22q11.2” highlights its correlation with DiGeorge Syndrome. Interestingly, when searching “autism” and “schizophrenia”, we found the top ranking CNVs were similar, including 22q11.2, 15q11.2, 15q13.3, 1q21.1, 16p11.2, 3q29, 16p13.1, 16p13.11 and 17p12. Indeed, recent literature confirmed our finding that “autism” and “schizophrenia” are highly related. The finding from our database also suggested that both diseases shared a common genetic basis, which could be an interesting topic in neuroscience field.

### Finding diseases related to specified CNVs

Firstly, the utility of CNVdigest was exemplified by inquiring CNV “22q11.2”. (Fig. 4 and Fig. 5). 22q11.2 deletion is normally considered to be the cause of DiGeorge Syndrome, also known as velocardiofacial syndrome (VCFS) or CATCH 22 [25]. The key features include cardiac abnormality, abnormal facies, thymic aplasia, cleft palate, and hypocalcemia [3]. By searching 22q11.2 in cnv.gtxlab.com, a pie-chart and a list of disorders were returned. To our surprise, DiGeorge Syndrome is the second most-mentioned disease in previous literature, while schizophrenia being the 1st in the rank. Though inconsistent name usage (e.g. DGS, VCFS, etc.) of DiGeorge Syndrome was part of the reason of declined rank. The relation between 22q11.2 and schizophrenia is still worth noting. Actually, if we dig deeper, OMIM page of DiGeorge Syndrome (#188400) indeed suggest the relation by mentioning “schizophrenia” multiple times.

### Finding CNVs related to specified diseases

**Fig. 6:**
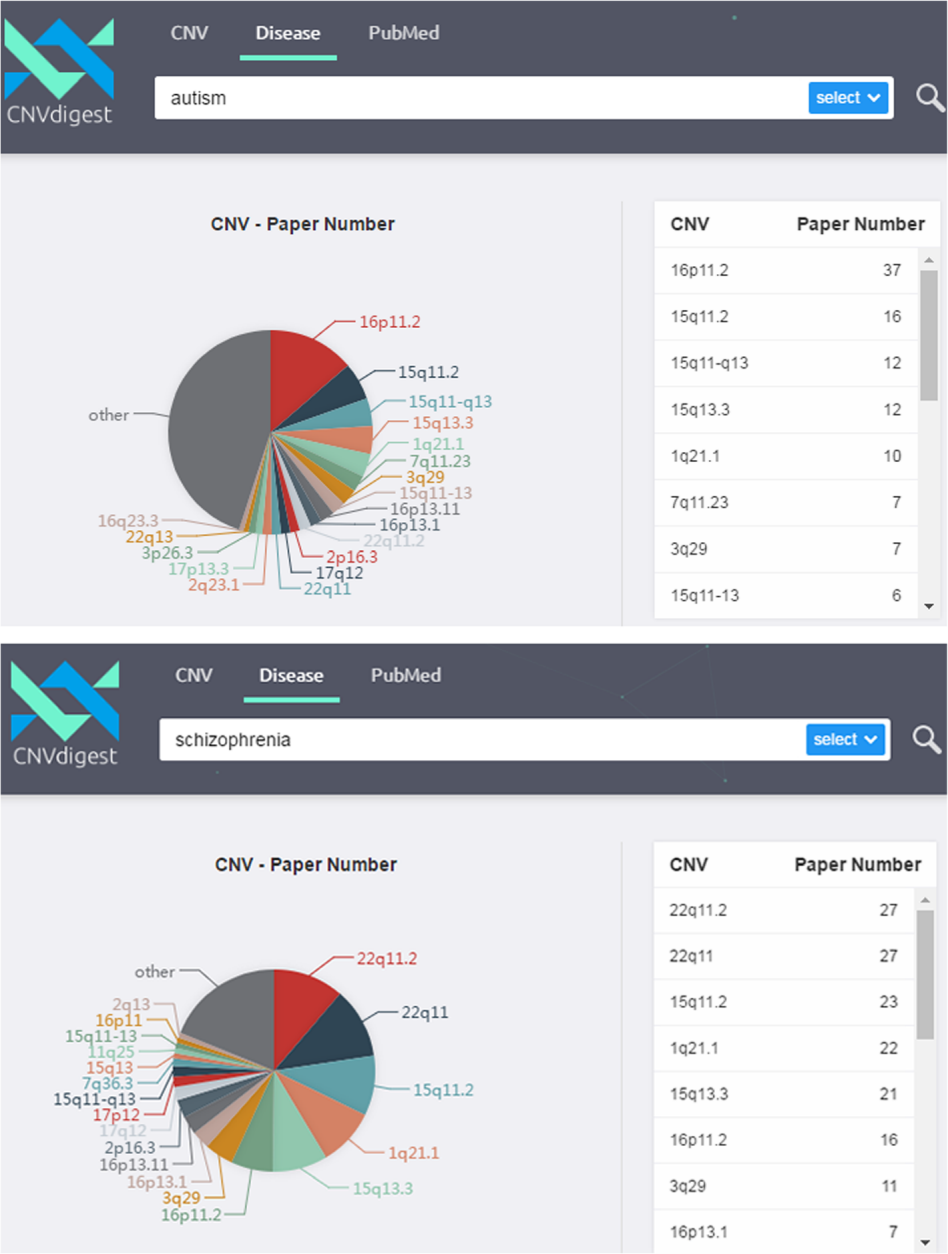
The returned results of searching diseases “autism” and “schizophrenia” in CNVdigest. The found cytobands and the numbers of paper were displayed with a pie-chart (left) and a list (right)

We demonstrate the utility of CNVdigest by searching “autism” and “schizophrenia”. (Fig. 6) These two disorders account for a big portion of CNV testing because of their high prevalence in the population. Interestingly, when we input “autism” and “schizophrenia” respectively in CNVdigest (cnv.gtxlab.com), nine of the top ten returned cytobands were the same: 22q11.2, 15q11.2, 15q13.3, 1q21.1, 16p11.2, 3q29,16p13.1,16p13.11 and 17p12. (Table 1). Previous literature already discussed the association between autism and schizophrenia [26, 27]. With CNVdigestwe further confirmed the linkage of autism and schizophrenia by showing their association with a set of common chromosomal abnormalities.

**Table 1:** Autism and Schizophrenia share a common set of CNVs

Autism	Schizophrenia	The common CNVs
16p11.2	22q11.2	16p11.2
15q11.2	15q11.2	15q11.2
15q13.3	15q13.3	15q13.3
1q21.1	1q21.1	1q21.1
16p13.1	16p11.2	16p13.1
22q11.2	3q29	22q11.2
3q29	16p13.1	3q29
17q12	2p16.3	17q12
2p16.3	17p12	2p16.3
7q11.23	17q12	

Note: Top ten CNVs in the returned list from searching “autism” or “schizophrenia” were listed and compared. Note: for cytobands that overlaps, only one cytoband is listed, e.g. 16p13.1 is listed when both 16p13.1 and 16p13.11 are shown

As autism and schizophrenia are both cognitive disorders, which might be attributed to defects in neuron development due to the CNV caused genetic variations, to further analyze the common CNVs, the genes in the affected regions, and the pathways containing the genes will facilitate our understanding of the pathogenic mechanisms of both diseases. The common CNVs also provide a candidate list for neuronal geneticists to study the neuron development and cognition formation; this list also provides a red-flag to clinic geneticists for a better differential diagnosis.

## Discussion

With the widely-spread use of array-based comparative genomic hybridization and next generation sequencing (NGS) copy number variant calling, the identification of CNVs became easier and easier. The ACMG Practice Guidelines recommended aCGH as the first-tier test for patients with developmental delay and intellectual disability, congenital anomalies, and dysmorphic features. Several online databases to catalogue and search for CNVs in normal and/or disease populations were developed to facilitate the CNVs interpretation in a clinical setting.

Database[32, 33] of Genomic Variants (DGV) (http://dgv.tcag.ca/dgv/app/home) provides a comprehensive summary of CNVs from the general population. The Clinic Genome Resource (ClinGen) [3] (www.clinicalgenome.org/) provides an authoritative central resource that defines the clinical relevance of genes and variants for use in precision medicine and research, where clinicians and researchers can share knowledge to expedite the understanding of CNV in patients with a variety of diseases.

DECIPHER stands for Database of Chromosomal Imbalance and Phenotype in Humans using Ensembl Resources (https://decipher.sanger.ac.uk/syndromes#overview). It is a web service for the interpretation of CNVs’ medical relevance.

Usually, DGV-ClinGen-DECIPHER route is sufficient to interpret the CNV findings. All CNVs can be classified into five categories: pathogenic, likely pathogenic, variants of unknown significance (VOUS), likely benign or benign. In general, a CNV is interpreted as pathogenic or likely pathogenic if it resides on a chromosome locus that is listed in ClinGen, DECIPHER or internal database as pathogenic. A CNV is interpreted as benign or like benign if it localizes to a region that is listed in DGV or identical to ones detected in healthy family members.

Though the definition of pathogenic and benign is clear, it is not always easy to match the exact definitions. For all other CNVs that are not clearly matched can be seen as VOUS. The clinical relevance of VOUSs will be evaluated by literature search on PubMed. This step can be time consuming and labour intensive, as most relevant information can be buried in various details. CNVdigest provides a solution to display the most wanted information in a structured way, which can be a great complement to existing databases.

CNVD [28] was generated via manual text mining, which means a lot of laborious manual curation. Consequently, it was only able to include 6301 articles. Our system includes 49,422 abstracts of CNV-related articles (by June 2018). The key behind this massive number is the adoption of automatic text-mining methods, which include named entity recognition (both CNVs and diseases) as well as relation extraction. A comparison of numbers is listed in Table 2.

**Table 2:** comparison with an existing similar database CNVD

		CNVD	CNVdigest
Original data		6301 articles	49,422 abstracts
Methods		Manual Methods	automatic text-mining
Number of CNVs		unknown	1582
Number of Diseases		792	2425
Page display	Relation	Rough	Precise (trigger words)
	Original sentence	NULL	Displayed
	polarity descriptions	NULL	Displayed (duplication/deletion)

In addition, in order to help researchers to obtain the literature context, we provide the evidence sentences for each CNV-disease pair.

The articles in our system only include abstracts. This is due to two reasons: (1) the availability of full-texts is limited; (2) the performance of state-of-the-art text mining tools could drop significantly when run on full-texts instead of abstracts.

Some trial users of our system proposes the need for automated machine translation. For instance, clinical doctors might want to refer to the facts in system presented in Chinese. This is a challenging task, as it requires automated machine translation and a comprehensive mapping from English biomedical terms to terms in desired languages. We will make it a priority for future development and hopefully introduce it in following updates of the system.

## Conclusions

The interpretation of the relationship between CNVs and diseases is of great clinical importance in genetic testing. A massive amount of such information is buried in literature. We employed state-of-the-art text mining methods, integrated a number of components to perform the relation extraction of CNVs and diseases by harnessing the computational power of a supercomputer. The resulting system, CNVdigest, is a web-based system performing integrative human copy number variant (CNV) analysis. It identifies medical associations between CNVs and diseases according to literature evidences. The webserver integrates information extracted from all searchable published literatures on NCBI that mentioned CNVs. With a concise front-end, CNVdigest can simplify the analyses of CNV/disease association. Users can use CNV as an input to search for the associated diseases, and vice versa. Conveniently, users can apply CNVdigest to understand detected CNVs from clinical genetic tests (like those generated by NIPT-CNV-CLOUD, another webserver we developed). We demonstrate the utility of CNVdigest exemplified by inquiring CNV “22q11.2” and searching diseases “autism” and “schizophrenia”.

## Abbreviations

CNV: Copy number variant
NER: Named entities recognition
RE: Relation extraction
